# Beyond Field Effect: Analysis of Shrunken Centroids in Normal Esophageal Epithelia Detects Concomitant Esophageal Adenocarcinoma

**DOI:** 10.4137/bbi.s311

**Published:** 2009-11-24

**Authors:** Florin M. Selaru, Suna Wang, Jing Yin, Karsten Schulmann, Yan Xu, Yuriko Mori, Alexandru V. Olaru, Fumiaki Sato, James P. Hamilton, John M. Abraham, Paul Schneider, Bruce D. Greenwald, Jan Brabender, Stephen J. Meltzer

**Affiliations:** 1 Gastroenterology Division, Department of Medicine, the Johns Hopkins University School of Medicine, Baltimore, MD 21231; 2 Gastroenterology Division, Department of Medicine, University of Maryland School of Medicine and Baltimore VA Hospital and Greenebaum Cancer Center, Baltimore, MD 21201; 3 Division of Gastroenterology, Department of Medicine, Ruhr-University Bochum, Bochum, Germany; 4 Department of Visceral and Vascular Surgery, University of Cologne, Germany

## Abstract

**Background and Aims:**

Because of the extremely low neoplastic progression rate in Barrett’s esophagus, it is difficult to diagnose patients with concomitant adenocarcinoma early in their disease course. If biomarkers existed in normal squamous esophageal epithelium to identify patients with concomitant esophageal adenocarcinoma, potential applications would be far-reaching. The aim of the current study was to identify global gene expression patterns in normal esophageal epithelium capable of revealing simultaneous esophageal adenocarcinoma, even located remotely in the esophagus.

**Methods:**

Tissues comprised normal esophageal epithelia from 9 patients with esophageal adenocarcinoma, 8 patients lacking esophageal adenocarcinoma or Barrett’s, and 6 patients with Barrett’s esophagus alone. cDNA microarrays were performed, and pattern recognition in each of these subgroups was achieved using shrunken nearest centroid predictors.

**Results:**

Our method accurately discriminated normal esophageal epithelia of 8/8 patients without esophageal adenocarcinoma or Barrett’s esophagus and of 6/6 patients with Barrett’s esophagus alone from normal esophageal epithelia of 9/9 patients with Barrett’s esophagus and concomitant esophageal adenocarcinoma. Moreover, we identified genes differentially expressed between the above subgroups. Thus, based on their corresponding normal esophageal epithelia alone, our method accurately diagnosed patients who had concomitant esophageal adenocarcinoma.

**Conclusions:**

These global gene expression patterns, along with individual genes culled from them, represent potential biomarkers for the early diagnosis of esophageal adenocarcinoma from normal esophageal epithelia. Genes discovered in normal esophagus that are differentially expressed in patients with *vs*. without esophageal adenocarcinoma merit further pursuit in molecular genetic, functional, and therapeutic interventional studies.

## Introduction

One of the greatest challenges in the management of Barrett’s esophagus (BE), the precursor lesion of esophageal adenocarcinoma (EAC), is to expeditiously identify patients who have early EAC and to predict those who are most likely to develop EAC. The rate of progression to cancer (0.4%–1% per year) is very low, making this challenge particularly difficult (Reynolds et al. 1999; Cameron, 1997). Moreover, in the surveillance of BE, a meticulous endoscopic search is often performed to histologically identify grossly normal-appearing dysplastic or cancerous lesions. However, the value of this type of systematic surveillance has been questioned, due to its low sensitivity and specificity (Conio et al. 2003). Thus, from a purely practical standpoint, it would be advantageous to be able to identify patients with malignant esophageal lesions simply by biopsying their normal squamous esophagus.

The presence and degree of dysplasia constitute the most widely accepted measure of neoplastic risk in Barrett’s esophagus. However, significant problems have emerged demonstrating the need for improved progression risk biomarkers. These problems include poor interobserver reproducibility of dysplasia interpretation and inconsistent rates of progression as well as regression of dysplasia, both of which have made it difficult to develop national surveillance guidelines (Rana and Johnston, 2000; Conio et al. 2003; Reid et al. 2000). Flow cytometry has shown promise in detecting a subset of patients who do not have high-grade dysplasia (HGD) but do have an increased risk of progression (Reid et al. 2000). The human genome project has yielded high-throughput methodologies for the computer analysis of data, which provide volume and quality control required to select clinically useful biomarkers (Taramelli and Acquati, 2004; Varmus and Stillman, 2005; Yoshida, 1999). 17p (p53)-loss of heterozygosity (LOH) has also shown potential as a molecular biomarker (Reid et al. 2003). In addition, methylation of p16, IN × 3 and HPP1 have been shown to predict progression to HGD and EAC (Hardie et al. 2005) (Geddert et al. 2004) (Schulmann et al. 2005). Molecular alterations have been found in Barrett’s metaplasia which reveal a field effect in premalignant metaplastic mucosa, but not in normal epithelium. For example, aneuploidy and loss of heterozygosity have been observed in metaplastic mucosa from Barrett’s patients with dysplasia or adenocarcinoma (Blount et al. 1993; Boynton et al. 1991; Raskind et al. 1992; Reid et al. 1987). Similarly, p53 tumor suppressor gene point mutations have been reported in Barrett’s metaplasia (Casson et al. 1994; Huang et al. 1993; Meltzer et al. 1991), and altered promoter DNA methylation has also been described for some tumor suppressor genes in Barrett’s esophagus (Eads et al. 2000a; Kawakami et al. 2000; Klump et al. 1998; Wong et al. 1997). In contrast, most published studies to date report no DNA alterations (e.g. point mutations, methylation, or loss of heterozygosity) in normal squamous esophageal epithelium from patients with esophageal cancer. Corn et al. (Corn et al. 2001) reported E-cadherin methylation in Barrett’s esophagus specimens and esophageal adenocarcinoma, but not in normal esophageal epithelium. Another study showed that the expression of a panel of 23 genes capable of differentiating between Barrett’s esophagus and esophageal adenocarcinoma was unable to distinguish between the normal epithelia of Barrett’s metaplasia and adenocarcinoma patients (Brabender et al. 2004). One notable exception was the study by Eads et al. which found methylation of the CALCA, MGMT, and TIMP3 genes in the normal esophagus of a subset of patients with Barrett’s-associated esophageal dysplasia and adenocarcinoma (Eads et al. 2001). cDNA microarrays promise more accurate prediction than do classical clinical diagnostic tools (such as histologic categorization). However, the main challenge posed by microarrays is to construct meaningful classifiers based on gene expression profiles, using appropriate bioinformatics tools. A number of bioinformatics tools have been proposed, including artificial neural networks (Selaru et al. 2002a), hierarchical clustering (Selaru et al. 2002b; Zou et al. 2002) and principal components analysis (Mori et al. 2003; Selaru et al. 2004). In this paper, we have used shrunken nearest centroid predictors (SNCPs), an analysis technique adapted from classical nearest centroids predictors to gene microarray analysis (Tibshirani et al. 2002). The aim of the current study was to identify global gene expression patterns as well as individual genes in normal esophageal epithelium capable of revealing simultaneous esophageal adenocarcinoma, even located remotely in the esophagus.

## Materials and Methods

### Patients and tissues

Six patients with BE alone, 9 with BE and concomitant EAC, and 8 with neither BE nor EAC were included in this study. The 8 patients without BE or EAC had had endoscopy for unrelated indications, such as peptic ulcer disease, but had undergone endoscopic biopsy of the gastroesophageal junction that was histologically normal. In all cases, biopsies from grossly normal-appearing squamous esophageal epithelium at least 7 cm proximal to the upper limit of the Barrett’s mucosa were included in the study. None of the patients with BE alone had concomitant dysplasia. Fresh NE (normal esophagus) biopsy specimens were immediately frozen and stored in liquid nitrogen until further use. Matching morphologic controls were obtained from the same sites as the research specimens and were examined by hematoxylin and eosin staining by an expert gastrointestinal pathologist at the University of Maryland. Informed consent was obtained from all patients under an institutionally approved research protocol.

### Location of the normal squamous esophageal biopsies

The NE areas biopsied were grossly normal, without any endoscopic evidence of esophagitis or reflux changes. In patients with obvious mass lesions in their esophagus, biopsies were obtained at least 7 cm proximal to these lesions. Similarly, biopsies from BE patients were performed at last 7 cm away from the area that showed endoscopic evidence of Barrett’s esophagus. In patients lacking BE or EAC, biopsies were performed from areas that did not show any gross endoscopic abnormalities. Finally, all NE specimens were analyzed histologically, and there was no evidence of any metaplasia or other changes found in any of these NE samples.

### cDNA microarray production and hybridization

Detailed protocols for glass slide coating, cDNA clone preparation and verification, microarray printing, post-printing slide processing, RNA extraction, RNA amplification, labeling and hybridization have been published by our laboratory (Selaru et al. 2002b; Xu et al. 2002; Zou et al. 2002).

### RNA Extraction, Amplification, and Labeling of the aRNA Probe

Total RNA (3–20 μg) was extracted from freshly frozen tissue using an RNeasy kit (Quiagen, Valencia, CA) and amplified using the AmpliScript T7-flash transcription kit (Epicentre, Madison, WI). Labeling was performed on 6 μg of aRNA by incorporating Cy3- or Cy5-labeled dCTP using random primers and Superscript reverse transcriptase (Xu et al. 2002). The resulting probes were purified with a Microcon microcentrifuge filter device and recovered in a volume of 25 μl. The reference probe was prepared from an equimolar mixture containing aRNAs from eight human malignant cell lines, as described previously. Microarray preparation was performed as described (Selaru et al. 2002b; Xu et al. 2002; Mori et al. 2003).

### Microarray normalization

We adapted an algorithm for normalizing microarray data that improves its accuracy and dynamic range (Yang et al. 2002). Both within-slide and inter-slide normalization were accomplished: in this fashion, local distortions in signal and background intensity within different regions of a slide, as well as overall differences in hybridization or labeling efficiencies between slides, were overcome. We determined that our within-slide normalization performs optimally when 4 blocks are used as the normalization unit (each block being produced by a different microarray pin). We assumed that each group of 4 blocks was equivalent in average signal intensity and range to the next group of 4 blocks on the array. Thus, we utilized 8 normalization units per slide. This assumption was based on an optimization strategy in which we tested groups of 1, 2, 4, 8, and 16 blocks as the normalization unit, which showed that the 4-block unit performed with the least inaccuracy when a random number generator was used to produce the 8,064 values on a microarray slide (data not shown). Thus, this normalization method (Yang et al. 2002) consisted of 3 steps: intensity-dependent normalization within each slide, scale normalization within each slide, and inter-slide normalization.

### Shrunken nearest centroid predictor (SNCP) model

This method is an adaptation of classical nearest centroids prediction analysis, tailored specifically to microarray data (Tibshirani et al. 2002). Each centroid is comprised of weighted averages of genes (elements) on the microarray for a particular diagnostic category, or “class.” Thus, the centroids each contained 8,064 elements, since there were 8,064 genes on each microarray. Gene weighting was directly proportional to the raw average expression value, but inversely proportional to the standard deviation (i.e. the variability) of expression value within a given class. Centroids were then shrunken by adjusting the *threshold* value, which removed genes with lower weighted averages (thus yielding a smaller set of relevant genes). Gene expression variations below a certain *threshold* value were made equal to zero and ignored. Thus, shrinkage consisted of moving the centroid towards zero by threshold, and setting it equal to zero when it drops completely (Tibshirani et al. 2002).

The choice of Δ (amount of shrinkage) was dependent upon 2 variables: 1. prediction error minimization; 2. the number of genes that are left in the model. More specifically, when all the genes on the microarrays are used, the prediction error is significant. During the process of data fitting, the SNCP model excludes outliers, i.e. genes that are not usable for the prediction. It is, however, possible to achieve the minimum prediction error for a range of Δs. In this particular case, the model can predict the predefined categories using a variable number of genes. Under these conditions, the smaller the Δ, the higher the number of genes left in the model, and *vice versa*.

Internal validation of results was performed using cross-validation. The value of K (fold cross-validation number) was set by default at 10; therefore, we performed a 10-fold cross-validation. In this 10-fold cross-validation, the specimens were randomly assigned to 10 groups. Nine of the ten groups were used for training, while the prediction is made on the 10th group. This procedure is repeated 10 times. For example, in the Normal-Normal *versus* Normal-Cancer comparison, training is done on 16 specimens, and then the model predicts the 17th specimen.

### Permutation analysis

SNCPs are mathematical models that learn by example. In other words, SNCPs identify a centroid for every group in the comparison. New specimens are classified by calculating the distance between the new specimen and each of the centroids. The specimen is classified into the class whose centroid is closest to the specimen. Ideally, the SNCPs should be tested on a test set, composed of specimens that were not used during training. This, however, may prove difficult when a small number of specimens are available for the study. One method to circumvent the need for a test set, while ensuring statistical significance, is permutation analysis.

Permutation analysis is a statistical technique used to calculate the chances of obtaining classification results purely by chance. The analysis consists of randomly permuting the specimen labels and constructing classifiers (SNCPs) to categorize the specimens. In the current study, permutation resulted in randomly assigning specimens to one of two categories: N-N (NE specimens from patients lacking EAC or BE) and BE-CA (NE specimens from patients with Barrett’s esophagus and concomitant esophageal adenocarcinoma). We subsequently chose the SNCP model with the lowest prediction error. We repeated this procedure 100 times. In all 100 random permutations, SNCPs were unable to learn the 2 categories correctly (with an error = 0). The mean group error for the 100 permutations was 0.36. This finding demonstrates that the possibility that *our* SNCP learned the 2 categories (N-N and BE-CA) correctly by chance alone was less than 1 in 100.

## Results

In the current study, we sought to determine whether the SNCP strategy could be used to identify gene expression patterns or individual genes as biomarkers to distinguish between the normal esophagus of patients with, *vs.* without, accompanying EAC. SNCPs discovered both broad patterns and individual genes that were highly accurate in their ability to identify whether or not a patient had accompanying remotely located cancer.

**Figure 1.**
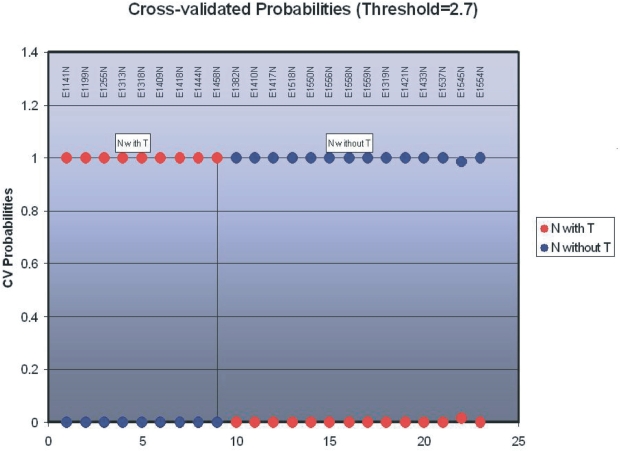
Predicted diagnoses in comparison of NE from EAC patients *vs*. patients with either BE only or no BE. Patients with EAC, to the left of and on the vertical line, were diagnosed correctly in every case, as were all control patients without any lesion (to the right of the vertical line). *Red*, likelihood of being an EAC patient; *blue*, likelihood of being a noncancer patient.

In our initial application of this strategy, we considered NE biopsy specimens of both subjects with completely normal esophagi (Normal-Normal, or N-N) and patients with BE but without EAC (BE-alone) together as a single group, which we compared to NE biopsy specimens of patients with Barett’s esophagus with concomitant EAC (BE-CA). Centroids were 100% accurate in predicting which subject or patient was in which group in this comparison, as shown in Figure 1. A list containing 195 genes was generated, based on their differential expression between normal esophagi from normal patients and normal esophagi from patients with EAC. Table 1contains a few of these genes, with already known links to cancer.

**Figure 2.**
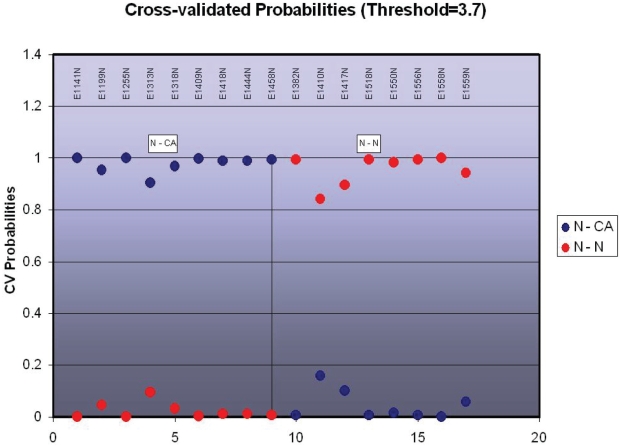
Predicted diagnoses in comparison of NE from EAC patients *vs*. control subjects (patients with neither BE nor EAC). Patients with EAC, to the left of the vertical line, were diagnosed correctly in every case, as were all control subjects patients without any lesion (to the right of the vertical line). *Blue*, likelihood of being a control subject; *red*, likelihood of being an EAC patient.

In an effort to further narrow the number of variables involved in the difference between NE biopsies from patients with concomitant EAC *vs.* subjects without EAC, we also compared NE from subjects without esophageal disease *vs.* NE from EAC patients only (i.e. excluding noncancer subjects with BE). This comparison revealed the accuracy of centroids in distinguishing these two subgroups, as shown in Figure 2.

**Figure 3.**
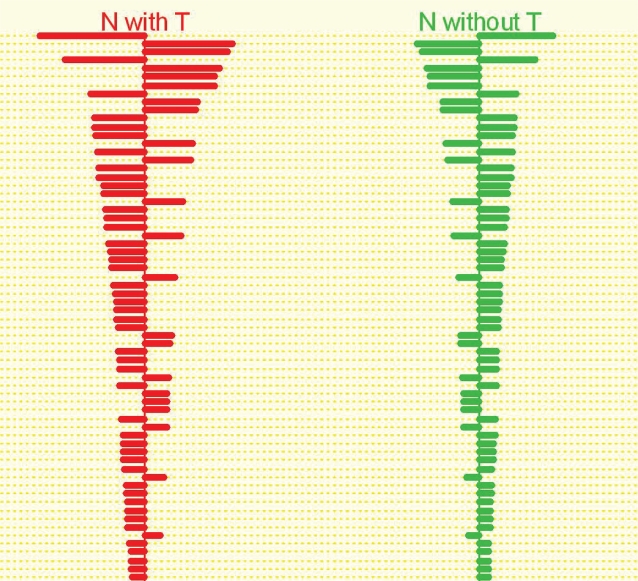
Centroids from comparison between NE of patients with *vs*. without accompanying EAC. Overexpressed genes are designated by *rightward*-extending bars; those that are underexpressed protrude to the *left. Red* centroid, NE specimens from subjects with EAC; *green* centroid, NE from patients without EAC. This plot demonstrates that genes underexpressed in noncancer patients are overexpressed in EAC patients, and *vice versa*. SNCP threshold = 2.7.

The SNCPs also generated visual displays of centroids, showing which genes were overexpressed and which were underexpressed in NE from patients with *vs.* those without accompanying EAC. The genes in these displays are arrayed in order of decreasing differential expression, with the most differentially expressed genes at the top and the least differentially expressed genes at the bottom. One such typical centroid is displayed in Figure 3.

Genes represented in a shrunken centroid derived by comparing NE tissues between cancer and non-cancer patients are shown in Table 1. Among them are many genes with previous links to esophageal cancer or to cancers in general: histone biomarkers, gravin, HLA-DRA, keratin 8 (KRT8), glutathione peroxidase 2 (GPX2), the mitotic checkpoint protein kinase BUB1B, the progestin-induced protein DD5 and transglutaminase 3.

## Discussion

The rate of progression to cancer among Barrett’s cohorts (0.4%–0.5% per year in some studies, 0.5 to 1% per year in other studies) is small, making it highly important to identify patients who will imminently or ultimately develop cancer (Reynolds et al. 1999; Cameron, 1997). Moreover, sampling error may entirely miss molecular or histologic changes that occur only in Barrett’s metaplastic mucosa. Thus, it would be very useful to be able to identify patients with concurrent esophageal neoplastic progression merely by sampling their normal esophagus.

Previous studies have compared gene expression patterns among normal, metaplastic, and cancerous esophageal epithelia (Guillem et al. 2000; Lu et al. 2001; Selaru et al. 2002b; Xu et al. 2002). Moreover, a recent study by Wang, S et al. suggested that gene expression patterns in Barrett’s esophagus are significantly closer to gene expression patterns in esophageal adenocarcinoma than to expression patterns in normal esophagus. This finding somewhat alarmingly implies that Barrett’s esophagus is biologically closer to cancer than to normal esophagus (Wang et al. 2006). However, these studies have consisted of direct comparisons of these different types of esophageal epithelia to each other. In the current study, a different approach was undertaken: i.e., a comparison was made of the normal esophageal epithelia from patients at differing stages of esophageal neoplastic progression. This study found unique molecular signatures in normal esophageal epithelium that reflected concomitant neoplasia elsewhere in the esophagus.

The potential biologic ramifications of our study are far-reaching. The field effect found near esophageal tumors in surrounding normal epithelium has been well-described (Eads et al. 2000b; Eads et al. 2001). A recent study by Brabender et al. (Brabender et al. 2005) identified a field effect by using a gene expression panel. In the current study, biopsies of normal esophagus were obtained at least 7 cm away from the tumor or Barrett’s esophagus. The current findings suggest that esophageal cancer exerts a greater influence on the normal esophageal epithelium than previously known or suspected. While molecular alterations in histologically normal squamous esophageal epithelium have previously been described adjacent to cancers, the current findings suggest that alterations in gene expression and gene expression pattern accompanying cancer can affect large portions of the normal squamous esophagus. We postulate that the development of esophageal adenocarcinoma is accompanied by widespread molecular phenotypic alterations that involve the entire normal squamous esophageal epithelium.

The SNCP-based approach applied in the current study offers a number of advantages over other analytic techniques. These include the ability to differentiate among multiple specimen groups; the potential for rapid translation to the clinical setting; a low likelihood of overfitting, yielding a low probability of erroneous diagnoses in new, independent datasets; and the capacity to yield a reduced number of diagnostic genes, which can themselves be developed as individual biomarkers as well as the basis for further molecular genetic studies (Tibshirani et al. 2002).

In the current study, genes positioned the highest in centroids discriminating normal tissues from noncancer *vs.* cancer patients were both interesting and relevant. For example, among the most highly ranked genes were members of the histone families (Table 1).

As single-gene predictors, histone biomarkers were accurate in distinguishing between accompanying cancer and its absence. Histones are basic nuclear proteins responsible for the nucleosome structure of chromosomal fibers in eukaryotes. Apart from promoter hypermethylation, modification of histone proteins is the second major component of epigenetic transcriptional control. DNA methylation and histone acetylation are integrally linked. Methylation is catalyzed by a family of DNA methyltransferases. DNA methyltransferases recruit histone deacetylases, leading to histone deacetylation and transcriptional repression. Methylated DNA is also recognized by a family of methylated DNA-binding proteins, which recruit histone deacetylases and ATP-dependent chromatin remodeling proteins, resulting in a tightly condensed chromatin structure and gene inactivation. Additional links between the “histone code” and the “cytosine methylation code” are increasingly evident (Johnstone, 2002; Kouraklis and Theocharis, 2002; Marks et al. 2001).

In addition, alterations of proteins in the histone acetyltransferase family (e.g. CREB-binding protein and p300) are associated with cancers of the breast, colon, liver, and hematopoietic system. Of particular relevance to the current findings, histone H4 is hyperacetylated in early stages of esophageal cancer cell invasion, and thereafter changes to a hypoacetylated state according to the degree of cancer progression (Toh et al. 2003). These results suggest that a dynamic equilibrium between histone acetylase and deacetylase activities is disrupted in esophageal carcinogenesis, implying that an interaction may exist between hyperacetylation of histone H4 and histone deacetylase 1 expression (Toh et al. 2003).

Similarly, by applying differential display to esophageal tumor and matched normal esophageal samples, histone H3.3 was identified among 49 cDNA ddPCR clones from esophageal cancers (ECs) (Graber et al. 1996). Histone H3.3 was overexpressed in 4/6 ECs, but not in paired normal mucosa. Only 5/13 normal human cell lines from various organs, but 11/12 human cancer cell lines (including 9 of 9 adenocarcinoma lines) overexpressed H3.3 (Graber et al. 1996). Histones H3 and H4 were deacetylated in gastric cancer cell lines showing aberrant methylation of CHFR, a mitotic checkpoint gene, suggesting a role for histone deacetylation in methylation-dependent gene silencing (Satoh et al. 2003).

Another gene identified in the current study was HLA-DRA. Major histocompatibility complex (MHC) molecules are of central importance in regulating the immune response against tumors. Loss of expression of HLA class II molecules on tumor cells affects the onset and modulation of the immune response through lack of activation of CD4^+^ T lymphocytes. In part, loss of expression is caused by mutations as shown for large B-cell lymphoma (Jordanova et al. 2003). A recent study found downregulation of HLA-DRA in invasive cancers compared to dysplastic cervical lesions (Chil et al. 2003).

We also observed a strong predictive value of keratin 8 (KRT8). KRT8 belongs to the intermediate filament family and associates with keratin 18 to form a heterotetramer of two type i and two type ii keratins. Its phosphorylation on serine residues is enhanced during EGF stimulation and mitosis. Dysregulation of keratin 8 is associated with esophageal carcinogenesis (Boch et al. 1997; Glickman et al. 2001a; Glickman et al. 2001b; Salo et al. 1996).

Additional genes with known relevance to human cancer identified by this SNCP model included glutathione peroxidase 2 (GPX2), the mitotic checkpoint protein kinase BUB1B, and the progestin-induced protein DD5. As expected, BUB1B was expressed at high levels in the normal esophageal tissues of patients without cancer and underexpressed in patients with cancer. BUB1B is a component of the mitotic checkpoint that delays anaphase until all chromosomes are properly attached to the mitotic spindle. In BRCA2-deficient murine cells, BUB1 mutants potentiate growth and cellular transformation (Davenport et al. 1999). In addition, mutations in human BUB1B have demonstrated a dominant negative effect by disrupting the mitotic checkpoint when transfected into euploid colon cancer cell lines (Davenport et al. 1999). Thus, BUB1B is a candidate tumor suppressor gene in the esophagus whose downregulation in normal esophageal tissue is associated with cancer development.

Transglutaminase 3, which was underexpressed in the normal tissue of tumor patients in our study, was recently found to be downregulated in esophageal squamous cell carcinoma and head and neck squamous cell carcinoma by cDNA microarray studies comparing cancer and matching normal tissue (Luo et al. 2003).

In conclusion, the current study diagnosed patients with remote esophageal neoplasia based on biopsies of their remote normal epithelium alone, and provided a minimal list of genes necessary to do so. This proof-of-principle study establishes a theoretical basis to identify cancers in other organs by studying gene expression patterns or other molecular signatures in their matching normal epithelia. In addition, by shrinking the number of genes needed to arrive at a correct diagnosis, the current work showcases an approach to identify smaller numbers of genes worthy of further research from microarray data, both as biomarkers and for biologic or functional studies.

## Grant Support

CA85069, CA95323, CA01808, CA106763, and CA77057.

## Figures and Tables

**Table 1.** Selected genes identified by comparison of NE from patients with EAC (N with T) *vs*. without EAC (N without T). Threshold value set at 2.7; **N with T**: gene score in the group of patients with esophageal adenocarcinoma; **N without T**: gene score in the group of patients without esophageal adenocarcinoma. Gene identifiers and gene names are shown in the two leftmost columns.

**Gene ID**	**Gene Name**	**N with T**	**N without T**
AB003476	gravin	−0.7322	0.4707
NM_005319	H1 histone family, member 2 (H1F2)	0.5907	−0.3797
XM_004416	H2A histone family, member L (H2AFL)	0.5384	−0.3461
NM_003519	H2B histone family, member C (H2BFC)	0.5062	−0.3254
NM_002273	keratin 8 (KRT8)	0.3834	−0.2465
NM_015902	progestin induced protein (DD5)	−0.3112	0.2001
NM_003516	H2A histone family, member O (H2AFO)	0.2322	−0.1493
XM_009572	transglutaminase 3 (TGM3)	−0.2078	0.1336
NM_019111	major histocompatibility complex, class II, DR alpha (HLA-DRA)	0.1695	−0.109
AF107297	mitotic checkpoint protein kinase BUB1B (BUB1B)	−0.0626	0.0402
NM_002083	glutathione peroxidase 2 (gastrointestinal) (GPX2)	0.0614	−0.0395

## Abbreviations

BE: Barrett’s esophagus;
EAC: esophageal adenocarcinoma;
NE: normal esophageal epithelium;
SNCPs: shrunken nearest centroid predictors;
BE-CA: normal esophageal epithelia from patients with Barrett’s esophagus and esophageal adenocarcinoma;
N-N: normal esophageal epithelia from patients lacking esophageal adenocarcinoma or Barrett’s;
BE-only: normal esophageal epithelia from patients with Barrett’s esophagus alone.
